# Therapeutic Plasma Exchange Practices in Intensive Care Unit

**DOI:** 10.5005/jp-journals-10071-23209

**Published:** 2019-07

**Authors:** Lakshmi Ranganathan, Rema Menon, Nagarajan Ramakrishnan, Ramesh Venkatraman, Pratheema Ramachandran

**Affiliations:** 1,3,4,5 Department of Critical Care Medicine, Apollo Hospitals, Chennai, Tamil Nadu, India; 2 Department of Transfusion Medicine, Apollo Hospitals, Chennai, Tamil Nadu, India

**Keywords:** Apheresis, Crystalloids, Plasma, Plasmapheresis, Therapeutic plasma exchange

## Abstract

**Objective:**

To observe the indications, practices and outcome of therapeutic plasma exchange (TPE) in a tertiary care ICU.

**Materials and Methods:**

The study involves retrospective analysis of 56 patients who underwent TPE between May 2011 and August 2013. Data relating to demographics, diagnosis, category of indication, number of sessions, volume and type of replacement solutions were collected.

**Results:**

Category I indications were 50%, with a mean of 3.32 sessions per patient. Per session volume exchanged was 9775.1 ± 11812.9 mL and replacement volume was 7414 ± 6993.03 mL. Fresh frozen plasma (FFP), crystalloids, cryopoorplasma and PRBC constituted 62.9%, 22%, 9.9% and 5.3% of volume replacement, respectively. TPE was terminated in three patients for Transfusion Associated Acute Lung Injury (TRALI), hypotension and cardiac arrest respectively. Clinical improvement was noted in 82% of patients and overall mortality rate was 12.5%.

**Conclusion:**

TPE is feasible and well tolerated in ICU with favorable disease resolution and outcome. Common indications included sickle cell and myasthenia crisis and blood products were the most commonly used for volume replacement.

**How to cite this article:**

Ranganathan L, Menon R, Ramakrishnan N, Venkatraman R, Ramachandran P. Therapeutic Plasma Exchange Practices in Intensive Care Unit. Indian J Crit Care Med 2019;23(7):336–338.

## INTRODUCTION

Therapeutic Plasma Exchange (TPE) is not an uncommon treatment modality used in ICU. Indications for TPE are wide and varied and some studies have demonstrated superiority of this therapy over intravenous immunoglobulin (IVIG).^1^ However, studies have demonstrated limitations of TPE primarily relating to adverse effects severe enough to consider cessation of therapy.^2^ In addition, TPE is feasible only in major referral centers as the procedure requires appropriate infrastructure, equipment and trained personnel. Moreover, in many settings crystalloids are utilized as a proportion of the replacement volume to minimize costs and the effect of such a strategy on outcomes is unclear.

In this descriptive study, we performed a retrospective analysis of patients undergoing TPE in our tertiary care unit with an objective to evaluate common indications, feasibility, tolerance, proportion of crystalloid replacement of plasma volume and outcome in different categories of indications.

## MATERIALS AND METHODS

### Study Population and Data Collection

We conducted a retrospective analysis of all patients (N = 56) who underwent TPE between May 2011 and August 2013. Data of demographic information, diagnosis, category of indication for TPE, number of TPE sessions, volume of plasma exchanged, type and volume of replacement solution infused, tolerance to TPE and outcomes in each category were analyzed. All procedures performed in the study were in accordance with the ethical standards of the institution and in accordance with Declaration of Helsinki.

### Categories of Indications for TPE

Patients were classified into 4 categories as per the 2010 guidelines of American Society For Apheresis (ASFA).^3^ Category I included indications where apheresis is accepted as a first line therapy; Category II when apheresis is accepted either as a solitary treatment or in conjunction with other treatment(s); Category III included disorders in which optimum role of apheresis therapy has not been established and Category IV when published evidence demonstrates ineffectiveness or harm from apheresis. The equipment used for plasmapheresis during the data collection period were continuous flow machines (Spectra optia-Manufacturer-Terumo BCT), based on the principle of differential centrifugation, in which patient details are entered and the inbuilt software algorithm suggests the appropriate volume of plasma to be exchanged and replacement fluid in each session.

## RESULTS

**Fig. 1:**
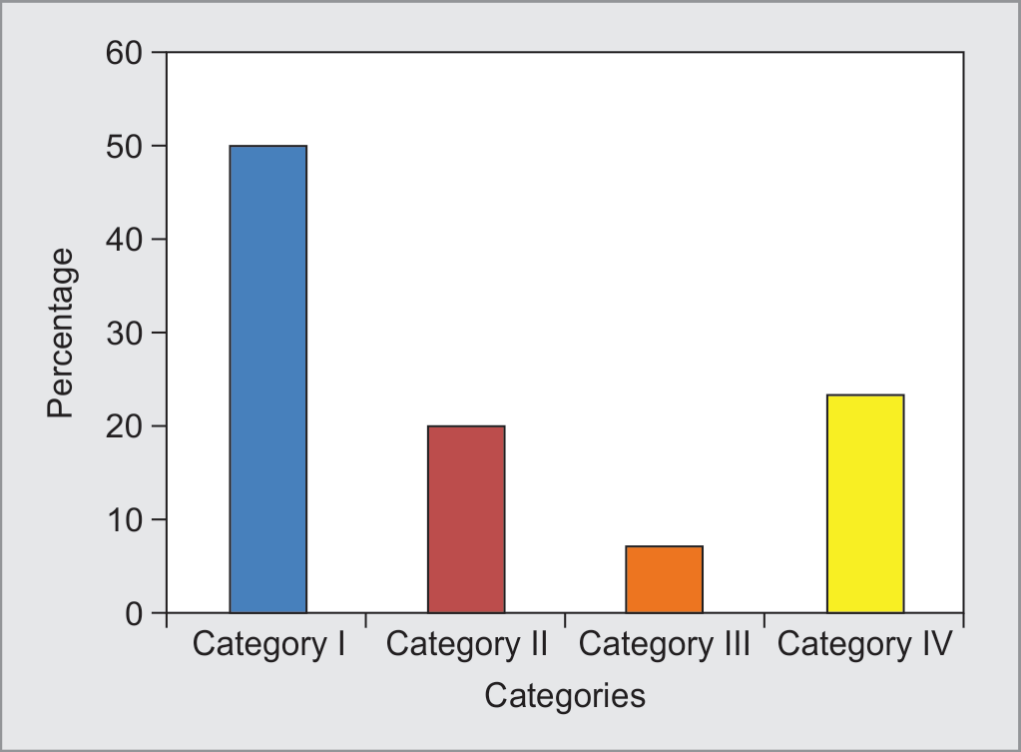
Category of patients as classified according to ASFA guidelines 2010

**Fig. 2:**
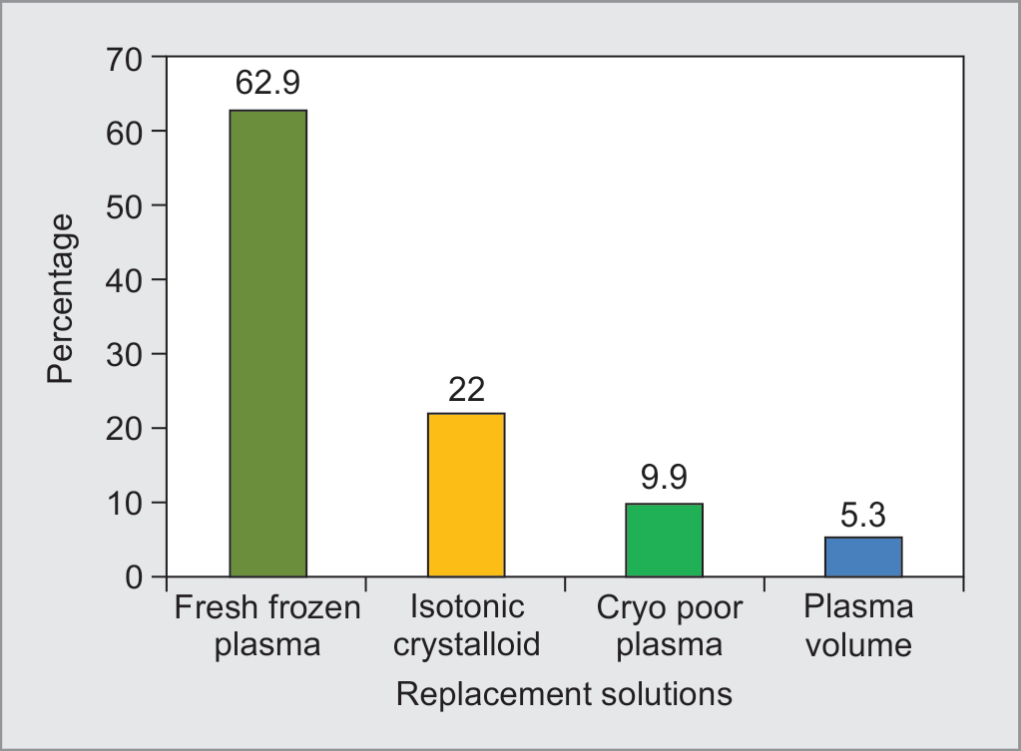
Volume exchanged and replacement solutions used

**Fig. 3:**
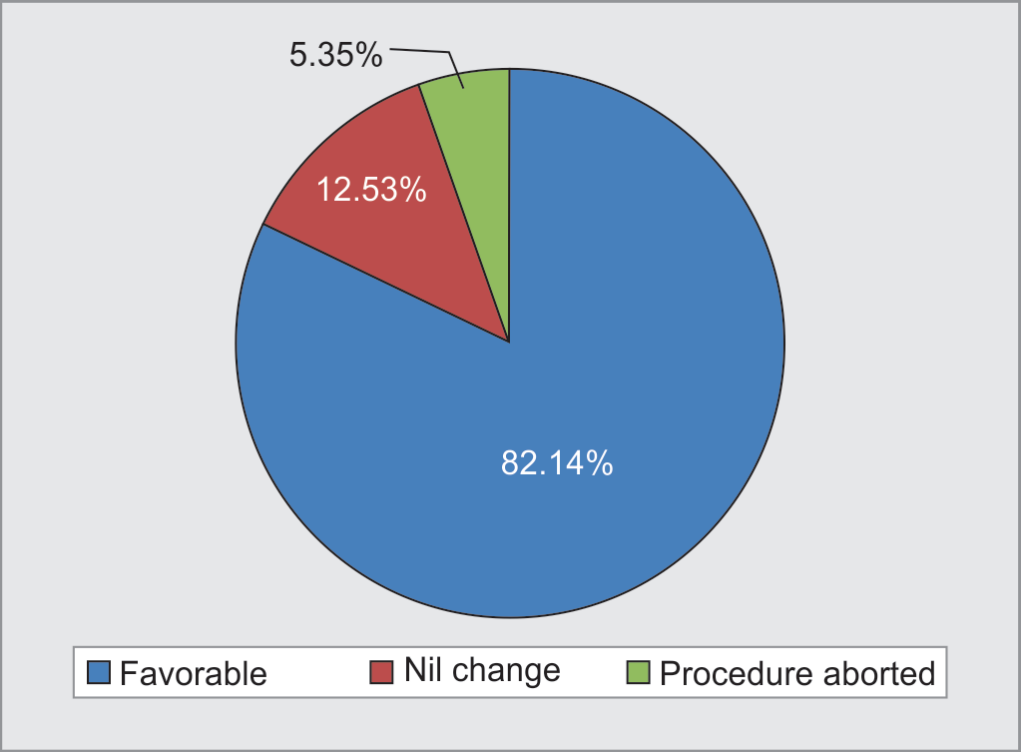
Outcomes of patients

A total of 56 patients were evaluated in our study including 33 males and 23 females (Table 1) with a mean age of 43.08 **±** 16.84. TPE was performed for category I indications in 50%, category II in 20%, category III in 7% and category IV in 23% patients (Fig. 1). The most common conditions were myasthenia Gravis, Guillain Barre Syndrome, Sickle cell anemia followed by Thrombotic Thrombocytopenic Purpura. In each category, total numbers of sessions, the mean plasma volume exchanged per patient, the mean replacement volume were analysed (Table 2). The volume exchanged was as per the standard prescription for the indication in each category, as recommended by ASFA guidelines.^3^ Fresh frozen plasma (FFP), isotonic crystalloids, cryo poor plasma and packed red cells constituted 62.9%, 22%, 9.9% and 5.3% of plasma volume replacement respectively (Fig. 2). TPE therapy was terminated before completion in three patients (Fig. 3). Indications for termination were development of transfusion related acute lung injury (TRALI), clinically significant hypotension and cardiac arrest. Clinical improvement was noted in 46 patients as judged by medical team providing care. Overall mortality rate in our study patients was 12.5%.

**Table 1:** Demographic details of patients in our study

*Total no of patients*	*56*
Gender	
Males	33 (58.93%)
Females	23 (41.07%)
Age (Mean ± Std.dev)	43.08±16.84
Outcome	
Survived	46 (82.1%)
Expired	7 (12.5%)
Lost to follow-up	3 (5.35%)

## DISCUSSION

In our single center observational study, we had used TPE for a variety of indications. Among these, the most common were myasthenia Gravis, Guillain Barre Syndrome (GBS), Sickle cell anemia followed by Thrombotic Thrombocytopenic Purpura (TTP), Systemic Lupus Erythematosus (SLE) and ABO incompatible transplantations. Earlier studies describing TPE practices in India^1^ and worldwide^4^ have suggested similar profile in both adult and pediatric patient population. Most studies have evaluated TPE use in specific patient population such as in ABO incompatible transplantations^5^ and GBS.^6^ Kaynar et al.^7^ in his retrospective multi-centre study in neurologic diseases observed that the mean number of sessions of TPE per patient was 5, and the mean processed plasma volume was 3,075 mL for each cycle. There was a good neurological improvement in a significant number of patients. In our study the mean number of sessions was lower likely because we evaluated TPE use for a variety of conditions, some of which may have responded faster than neurological illnesses. In our study we noted that 23% of treatments were done for Class IV indications. This likely indicates a tendency among practioners in our setting to use TPE on an experimental basis as salvage therapy even when evidence doesn't support it strongly. However, despite its use outside indications, we did not see significant intolerance or adverse effects.

**Table 2:** Details of TPE based on categories of indication

*Category*	*Category 1*	*Category 2*	*Category 3*	*Category 4*
Total number of patients	28	11	4	13
Average no of sessions	3.32	3.09	5.75	1
Volume exchanged per session (mL)	9775.1 ± 11812.9	7414 ± 6993.03	21763.7± 26647.3	154.6 ± 237.55
Replacement colloids per session (mL)	9122.9 ± 12464.24	6839 ± 7038.29	20138.5± 27199.03	70.7± 258.16
Replacement crystalloids per session (mL)	1098.2 ± 1431.62	227.2 ± 469.23	1450± 2396.52	202.3 ± 278.21
Expired	3	2	1	1
Lost to follow-up	1	1	1	0
Clinical improvement	24	8	2	12

The technique we used was similar to that used in other studies.^8^ The volume to be replaced was prescribed according to a standard formula^8^ as per ASFA guidelines. In most studies, volume of replacement solutions were about two times the plasma volume of the patients and average were 4 sessions.^9^

The type of replacement solutions used across various studies were variable. Studies had compared albumin and cryo-supernatant plasma with FFP. There was no statistical difference in benefits between the groups, but complications associated with blood components were more in the FFP group. Taking into account, the risks of blood component transfusion, and costs incurred crystalloids have become a major part of replacement fluids. In our study, 22% of plasma volume replacement was done using crystalloids with good clinical response.

Around 50 fatal reactions with TPE have been reported worldwide between 1978 and 1983.^1^ Of these, 16 were reported cardiac and 14 were respiratory related. Mortality has also been reported and attributed to anaphylaxis, sepsis and DIC. The complications noted in our study were TRALI in a myasthenic patient, hypotension possibly due to allergic reaction in a patient with vasulitis and cardiac arrest in a patient with mixed connective tissue disease following which the procedure was aborted. Similar outcomes of TPE related acute lung injury causing respiratory failure were reported by Kfoury Baz et al.^10^ The intolerance to TPE was minimal in our study. One likely reason could be the relatively lesser use of plasma in our patient population.

Our study has both strengths and limitations. The strengths were that we had a large database of a tertiary care unit where we could evaluate the objective clinical outcomes. Our study is also one of the very few studies that evaluated TPE practices across patients with several different diagnoses. We categorized the data using standard international criteria and our TPE prescription practices were based on ASFA guidelines. All patients were followed and outcomes were collected and reported comprehensively. The limitations of study are that it is a retrospective single center study. Exact details of patients’ weight-based volume exchange were unavailable and clinical improvement was not scored objectively since several indications were evaluated. We decided to take a pragmatic approach and recorded the outcomes based on the treating team's clinical judgment.

## CONCLUSION

TPE is feasible and well tolerated. Common indications for TPE in our study were sickle cell and myasthenic crisis.

## CLINICAL SIGNIFICANCE

Despite a higher proportion of crystalloid use as replacement volume favorable disease resolution was seen in most patients who underwent TPE.
